# A Comparison of Endodontic Microbiomes Associated With Symptomatic and Asymptomatic Apical Periodontitis by Next‐Generation Sequencing

**DOI:** 10.1111/iej.70140

**Published:** 2026-03-13

**Authors:** David Donnermeyer, Edgar Schäfer, Daniel Hagenfeld, Benjamin Ehmke, Karola Prior, Dag Harmsen, Madgalena Ibing, Sebastian Bürklein, Thomas Gerhard Wolf, Johannes Matern

**Affiliations:** ^1^ Department of Restorative, Preventive and Pediatric Dentistry, School of Dental Medicine University of Bern Bern Switzerland; ^2^ Department of Periodontology and Operative Dentistry University of Münster Münster Germany; ^3^ Central Interdisciplinary Ambulance in the School of Dentistry University of Münster Münster Germany

**Keywords:** asymptomatic apical periodontitis, microbiome, next‐generation sequencing, symptomatic apical periodontitis

## Abstract

**Aim:**

This cross‐sectional study aimed to compare the endodontic microbiome assessed from root canals of teeth associated with either symptomatic or asymptomatic apical periodontitis and analysed by 16S rDNA gene sequencing.

**Methodology:**

60 teeth presenting clinical and radiographic signs of symptomatic or asymptomatic apical periodontitis (*n* = 30) were included in this cross‐sectional study after participants had given their written informed consent. After isolation with rubber dam, disinfection and access cavity preparation, glide paths were prepared using C‐Pilot Files and K‐Files under electronic root canal length control. Microbial samples were collected from a total of 120 root canals (symptomatic apical periodontitis, SAP: *n* = 62, asymptomatic apical periodontitis, AAP: *n* = 58) each with a sterile file (size 20/0.06) in a single length technique. Only one specimen per tooth was included in the analysis; in multi‐rooted teeth, the specimen with highest sequencing depth. After DNA extraction, the hypervariable region V4 of the bacterial 16 S rRNA gene was amplified and sequenced (Illumina MiSeq). Taxonomy was assigned based on the expanded Human Oral Microbiome Database (eHOMD). Statistical analysis of diversity parameters comprised Mann–Whitney *U* tests and PERMANOVA. Compositional differences were evaluated by differential abundance analyses using DESeq2, LinDA, and ANCOM‐BC2 methods.

**Results:**

No differences were observed in richness and diversity (Shannon diversity index) on the genus or ASV level (*p* > 0.05). According to PERMANOVA, SAP and AAP microbiomes did not differ significantly both on genus and ASV levels (*p* > 0.05). Among highly abundant genera, *Fusobacterium* was indicated to be more abundant in SAP samples whereas *Actinomyces* was more abundant in AAP samples.

**Conclusions:**

The expression of clinical symptoms in apical periodontitis does not appear to be determined by specific microorganisms but may instead reflect shifts of the relative abundance of the microbial community.

## 1 Introduction

Apical periodontitis is a reaction to a miscellaneous and highly individual multispecies infection of the root canal spaces (Siqueira and Rocas 2009). Besides interindividual and regional differences in endodontic microbiomes reported in the literature (Santos et al. 2011; Rocas et al. 2006), a correlation between the distribution of microorganisms in the endodontic biofilms and the expression of clinical symptoms has been widely described. However, results are heterogeneous (Manoil et al. 2020), and recent reviews have found only limited evidence for the association of certain microorganisms and symptoms of endodontic origin (Aboushadi et al. 2024; Alhadainy et al. 2023).

For instance, a study including root canal specimens from teeth associated with either asymptomatic apical periodontitis (AAP) and symptomatic apical periodontitis (SAP) found similar prevalence of certain species irrespective of the clinical presentation (Rocas et al. 2011). Another analysis comparing the intracanal microbiome of AAP and the microbiome in pus specimens from acute apical abscesses could not identify specific taxa associated with the clinical presentation, although the proportional composition of taxa differed significantly (Rocas and Siqueira 2018). Corroborating the results, Santos et al. reported a higher prevalence of *Fusobacteria* in pus specimens of acute cases compared to root canal specimens derived from AAP root canals (Santos et al. 2011). In another study, root canal microbiomes in SAP cases were associated with significantly higher proportions of *Porphyromonas* (de Brito et al. 2020). Overall, symptomatic primary infections of the root canal system may exhibit greater microbial diversity than asymptomatic infections (Tzanetakis et al. 2015).

Besides the limited evidence, a significant limitation of merging the clinical studies into a clinical assumption is the methodological heterogeneity of the studies. While the closed‐ended reverse‐capture checkerboard approach (Rocas et al. 2011; Rocas and Siqueira 2018) and 454 pyrosequencing (454 Life Sciences, Branford, CT, USA; Roche, Basel, Switzerland) (Santos et al. 2011; Tzanetakis et al. 2015) were applied in each two of the aforementioned studies, only one relied on Illumina MiSeq (Illumina, San Diego, CA, USA) for sequencing (de Brito et al. 2020). The differences in depth of the analyses allow for little comparison only. Furthermore, sample sizes of less than 10 specimens per group (Santos et al. 2011; de Brito et al. 2020) likely result in inferior statistical power. Given the subjective nature of pain perception (Fillingim 2017) and the inevitable assignment to either AAP or SAP according to the clinical findings, results derived from smaller samples could easily be distorted by misclassification. Moreover, most available evidence originates from Brazil, while comparable studies from other regions are scarce, raising the possibility of geographic disparities (Arias‐Moliz, Ordinola‐Zapata, et al. 2024).

Interactions between endodontic microbiome shifts and clinical symptoms are highly relevant and still poorly understood. In the endodontic microbiome of primary infections, only few of the more than 500 identified bacterial species in root canal infections play a dominant role (Siqueira and Rocas 2022b). Most of them seem to have an unimportant role in the endodontic microbiome as only few are predominantly and repetitively identified (Siqueira and Rocas 2022b). Therefore, it has been postulated, that bacterial associations and their relative composition may drive the transition from asymptomatic inflammation to an acute inflammatory response in the periradicular tissue (Rocas et al. 2011). However, based in the available data, it is not possible to reliably identify specific pathogens in the community responsible for this transition. Sufficient numbers of root canal microbiomes need to be investigated with modern techniques to identify or exclude such effects. If specific virulent bacterial compositions could be verified, this would open new doors for further exploring the reason for such development, namely changes caused by the local endodontic environment or factors being associated with the host.

Therefore, the aim of the present cross‐sectional study was to compare the endodontic microbiome of root canals associated with asymptomatic apical periodontitis (AAP) or symptomatic apical periodontitis (SAP) using a large sample size and a contemporary 16S rRNA sequencing‐based identification method. The null hypothesis was that no difference exists in the alpha and beta diversity between the microbiomes of root canals from teeth associated with symptomatic or asymptomatic apical periodontitis.

## 2 Materials and Methods

**FIGURE 1:**
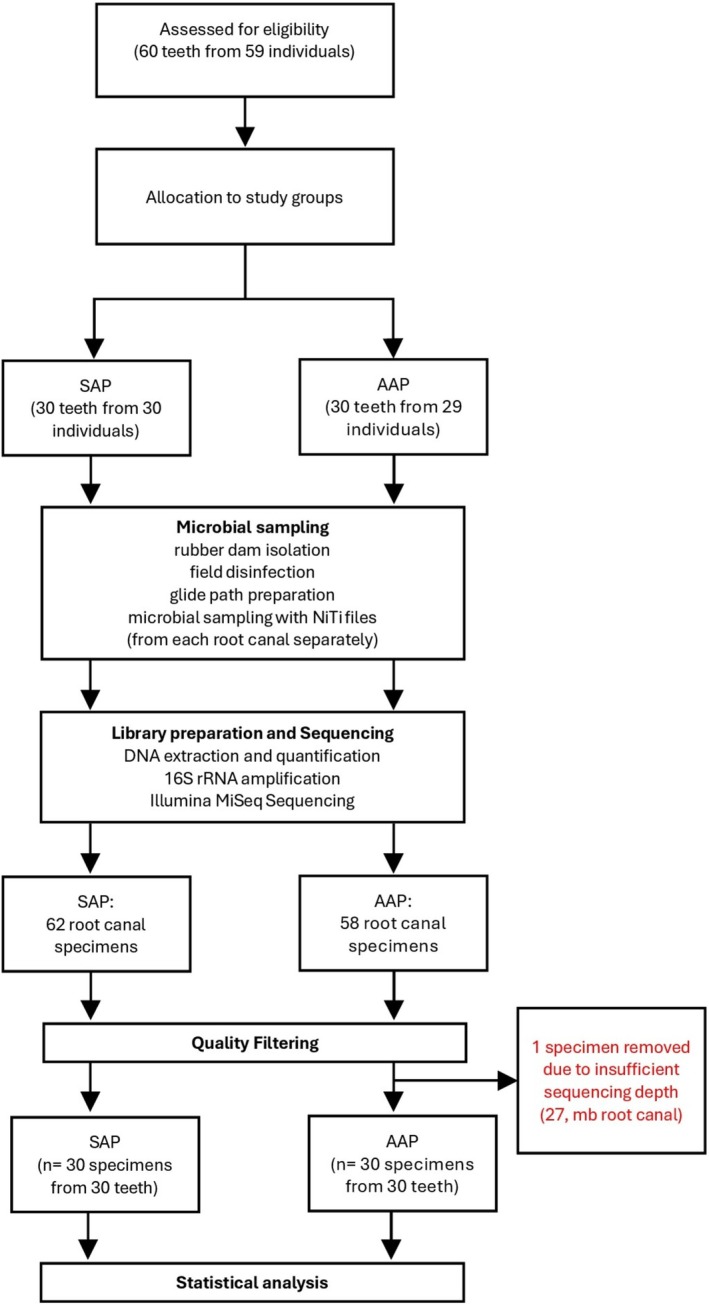
Flowchart illustrating the study design.

This observational study has been written according to Preferred Reporting items for Observational studies in Endodontics (PROBE) 2023 guidelines (Nagendrababu et al. 2023). A flowchart illustrating the study design is presented in Figure 1.

### 2.1 Sample Size Calculation

The sample size was calculated using the HMP R package (La Rosa et al. 2012), a multivariate statistic method, based on Dirichlet multinomial models via using data from the NIH Human Microbiome Project. For a three‐sample comparison targeted to compare microbiomes of symptomatic apical periodontitis, asymptomatic apical periodontitis, and asymptomatic apical periodontitis with fistula in further studies (Generalised Wald‐type test statistic; La Rosa et al. 2012) a power level of 0.9 could be achieved by computing 1000 Monte‐Carlo experiments setting alpha = 0.05, sequencing reads per sample = 50 000, sample size of *n* = 15 teeth per group. After collecting samples from 30 teeth, a post hoc power analysis to verify the adequacy of the sample size for the present 2‐sample comparison was conducted. Using the G*Power software, it was determined that the sample size achieves a statistical power of more than 99% to detect medium effect sizes (Cohen's *d* = 0.5). Thus, a sample size of 60 teeth per group was regarded sufficient for the present 2‐sample comparison. With regard to the possible overrepresentation of certain microbiomes from multi‐rooted teeth, the analysis was performed with the tooth as the unit of analysis. In this analysis, only one specimen per tooth was included and the specimen with the highest sequencing depth from a multi‐rooted tooth was chosen.

### 2.2 Study Population

This study was conducted at the Department of Periodontology and Operative Dentistry in the University Clinic Münster, University of Münster, Münster, Germany. The ethics committee of the medical association of Westphalia‐Lippe and the University of Münster approved a protocol for microbial sample collection from root canals and further investigation (2020‐180‐f‐S). All patients gave their informed written consent before participating in the study.

This cross‐sectional study comprised 60 teeth from patients between 18 and 75 years with age, gender and medical history recorded for all patients. The teeth presented clinical and radiographic signs of apical periodontitis and were further divided into the groups (*n* = 30) regarding the clinical type: symptomatic apical periodontitis (SAP) or asymptomatic apical periodontitis (AAP). Sequencing data of half of the teeth included in the present study were already analysed in a previous study (Donnermeyer et al. 2025) together with other specimens not included in the present study with a different objective, namely the comparison sampling techniques. Radiographic determination of apical periodontitis was defined as a periapical index of 3 or higher assessed by preoperative periapical radiographs (Orstavik et al. 1986). Participants were further assigned to one group according to the clinical findings. Clinical signs of apical periodontitis were defined as missing sensitivity to a cold stimulus (−45°C, Endo Cold Spray, Henry Schein, Melville, NY, USA) for all groups. Patients presenting without further symptoms were assigned to the AAP group whereas a tenderness to percussion or spontaneous pain were determinant for SAP. The assessment of clinical symptoms together with the clinical examination were performed in the same session with the microbial sampling procedures.

Exclusion criteria were as follows: patients reporting intake of antibiotics in the preceding 7 days before sample collection, patients requiring antibiotics before dental treatment, patients being pregnant or breastfeeding, patients with cognitive deficits, teeth with radiographic signs of root resorption, teeth with previously initiated root canal treatment or connection of the endodontic spaces to the oral cavity, teeth presenting with sinus tracts or associated swelling of the mucosa.

### 2.3 Microbial Sampling

Initially, each tooth was isolated with a rubber dam and the crown was disinfected applying sodium hypochlorite (3%, Hedinger, Stuttgart, Germany) for 1 min (Ng et al. 2003). Hereafter, the operative field was rinsed with water and an access cavity was prepared with sterile diamond burs and rose burs. After accessing the pulp chamber and the root canal orifices, a glide path was prepared with size 10 and 15 files (C‐Pilot #10, VDW, Munich, Germany; K‐File #15, Dentsply Sirona, York, PA, USA) under electronic root canal measurement control (RayPex 6, VDW). No irrigation of the pulp chamber and the root canal system was performed before sampling was completed. Hand instruments were used in one canal only and discarded afterward. Teeth which did not allow a glide path reaching the apical foramen to be established were excluded from the study. In each root canal, a sterile nickel‐titanium (NiTi) file (F6 SkyTaper, size 20/0.06, Komet, Lemgo, Germany) was inserted to full working length in a crown‐down approach for root canal preparation (single length technique) and extraction of biofilm together with root dentine in the instrument's flutes. The instruments were collected separately in sterile transport tubes (Safe‐Lock Tubes) containing 500 μL of sterile 10 mM Tris buffer (Sigma‐Aldrich, St. Louis, MO, USA) and the samples were either transferred to the laboratory directly for analysis or stored at −20°C until further processing. For each tooth, a routine root canal treatment was carried out after microbial sampling.

As described previously, controls for DNA extraction (*n* = 5), controls for library preparation (*n* = 10), and tris buffer containing new sterile NiTi instruments (*n* = 3) as well as mock samples as positive controls and to evaluate sensitivity and specificity were employed in each sequencing run to identify contamination during the process (Donnermeyer et al. 2025).

### 2.4 Library Preparation and Sequencing

Bacterial genomic DNA was extracted and purified using the QIAamp DNA Mini Kit (QIAGEN, Hilden, Germany) according to the manufacturer's protocol and the Qubit 2.0 fluorometer applying the Qubit dsDNA HS Assay Kit (Thermo Fisher, Waltham, MA, USA) served to quantify DNA concentration.

Library preparation was carried out according to the Illumina 16S metagenomics library preparation guide with one modification. Forward primer 515F‐Y 5′‐TCGTCGGCAGCGTCAGATGTGTATAAGAGACAG**GTGYCAGCMGCCGCGGTAA**‐3′ (Parada et al. 2016) and reverse primer 806R 5′‐GTCTCGTGGGCTCGGAGATGTGTATAAGAGACAG**GGACTACNVGGGTWTCTAATC**‐3′ (Klindworth et al. 2013) were applied for amplification of the V4 region of the 16S rRNA gene (16S rRNA gene specific nucleotides (nts) in bold). The Nextera XT Index Kit v2 (Illumina) was used to add sequencing adapters and sample specific dual indices.

Libraries were normalised and pooled in line with manufacturer's instructions. Sequencing was performed with the MiSeq Reagent Kit v2 in a 500‐cycles format producing 250 bp paired‐end reads. lllumina's MiSeq Control Software v.2.6.2.1, Real‐time Analysis Software v.1.18.54, and Reporter Software v.2.6.3 (Illumina) were applied for data acquisition and fastq generation.

### 2.5 Bioinformatic Pipeline

Searching for anchored primer sequences with a maximum error rate of 0.1, sequencing reads were adapter trimmed using Cutadapt (Martin 2011). Cutadapt processed sequencing reads comprise shortened reads with following lengths: 232 nts forward reads and 230 nts reverse reads. Sequencing data were processed and analysed with R (version 4.2.2; R Core Team 2022) and RStudio (version 2023.03.1 + 446; R Studio Team 2020). Exact amplicon sequence variants (ASVs) and output ASV tables were inferred applying the R package DADA2 (Callahan, Sankaran, et al. 2016). According to the DADA2 workflow, FASTQ files from each sequencing run were imported separately and, if not explicitly mentioned below, default settings were employed. The adapter‐ and index‐free reads were trimmed (the last 3′ nucleotide that did not contain a quality score was trimmed from each forward and reverse read, furthermore 23 nts from the forward reads and 21 nts from the reverse reads were trimmed at the 5′ end to compensate for single‐end overhangs). Hereafter, DADA2 trimmed reads were quality filtered. Reads with at least one “N” in the sequence (maxN = 0), a length shorter than 20 nts after truncation and trimming (minLen = 20) or more than two expected errors per read (EE) (maxEE = 2) were removed from the data set. Trimmed and filtered reads were denoised with the DADA2 algorithm and denoised data sets were merged to produce ASVs with maximally overlapping forward and reverse reads. Finally, chimeric sequences were removed, an ASV table was generated, and taxonomic assignment based on eHOMD data was performed (Chen et al. 2010).

### 2.6 Statistical Analysis

Using the phyloseq package in R (Mcmurdie and Holmes 2013), sample data were combined with the sequencing data in a phyloseq object. To reduce the influence of potential sequences on the intended analysis two more filtering steps were applied as follows: ASVs being observable in less than two samples and ASVs which were represented by less than 100 reads in the entire data set were eliminated. Samples and controls were separated into two data sets. A minimum of 10 000 reads per sample was regarded as an indicator of satisfying sample quality. Consequently, samples containing less than 10 000 reads would be removed from the sample data set. Using the vegan package of R to evaluate sufficiency of sequencing depth (Oksanen et al. 2022), rarefaction curves were calculated. Alpha diversity parameters such as richness, diversity (Shannon index), and dissimilarity (Bray–Curtis dissimilarity) were calculated following sample wise subsampling to even numbers of reads per sample to address differences in sequencing depth. A principal coordinate analysis (PCoA) based on Bray–Curtis dissimilarity and a permutational multivariate analysis of variance (PERMANOVA) using the vegan package of R to evaluate beta‐diversity differences between the groups (Oksanen et al. 2022) were performed. To check for relevant differences in the taxonomic composition differential abundance analyses (DA) on genus level were executed by using the R packages LinDA, ANCOM‐BC2, and DESeq2 (Love et al. 2014; Zhou et al. 2022; Lin and Peddada 2020). Prior to the differential abundance analysis, a prevalence filter was applied to remove genera that were prevalent in less than 20% from the data set. As low prevalent taxa cause high numbers of zeros appearing in the data set, which is still a challenge for differential abundance analysis methods, we decided to apply different differential abundance methods to increase robustness and confidence of results. The LinDA and ANCOM‐BC2 methods also allowed to employ a random effect model. The variable “patient” was defined as a random effect to account for the hierarchical structure due to the samples' heritage. The Benjamini & Hochberg (also known as “false discovery rate”) *p*‐value adjustment approach was used for addressing multiple testing. Only genera that showed consistent direction of effect size (log fold change) over all employed DA methods with at least one DA method indicating adjusted *p*‐value < 0.05 were considered differently abundant.

## 3 Results

60 teeth from 59 patients were included with each 30 teeth presenting with symptomatic or asymptomatic apical periodontitis. Of these 59 patients, 30 were female and 29 male (SAP: 17 female, 13 male; AAP: 13 female, 16 male; two teeth from the same male person). The mean age was 52.2 ± 13.8 years (SAP: 51.3 ± 15.2; AAP: 53.1 ± 12.4). The sample comprised 14 incisors, 19 premolars and 27 molars (SAP: 6/10/14, AAP: 8/9/13 (incisors/premolars/molars); more details in Table S1). Microbial samples were obtained from a total of 120 root canals, 62 from teeth associated with SAP and 58 from AAP cases. Only one specimen per tooth was included from multi‐rooted teeth when the specimen with the highest sequencing depth was chosen. The total number of raw reads per sample ranged from 16 063 to 836 589. After quality filtering, the number of retained reads per sample ranged from 11 623 to 596 148. One sample was removed from the dataset due to insufficient sequencing depth, with only 1098 raw reads and 53 retained reads after quality filtering. This specimen did stem from the mesio‐buccal root canal of a maxillary second molar (patient no. 58 in Table S1), while the other two specimens from the same tooth had sufficient sequencing depth. Sequencing depth and rarefaction curves are shown in Figure S1. The percentage of classified ASVs is presented in Table S2.

Alpha diversity parameters on genus level are given in Table 1. No differences were observed in the number of observed genera (richness) and diversity (Shannon diversity index) on genus level (*p* > 0.05). The same was observed for richness and diversity on ASV level (*p* > 0.05; Table S3).

**TABLE 1:** Alpha diversity parameters on genus level.

	SAP (*N* = 30)	AAP (*N* = 30)	*p*
Richness			
Mean (SD)	28.9 (12.4)	31.4 (14.3)	0.564
Median [Q1, Q3]	30.0 [19.5, 37.8]	29.0 [20.3, 42.0]	
Diversity			
Mean (SD)	1.94 (0.644)	1.91 (0.848)	0.764
Median [Q1, Q3]	2.04 [1.54, 2.42]	2.22 [1.29, 2.54]	

*Note:* Richness, Number of observed genera; Diversity, Shannon diversity index. Unpaired Mann–Whitney *U* tests.

### 3.1 Genus‐Level

**FIGURE 2:**
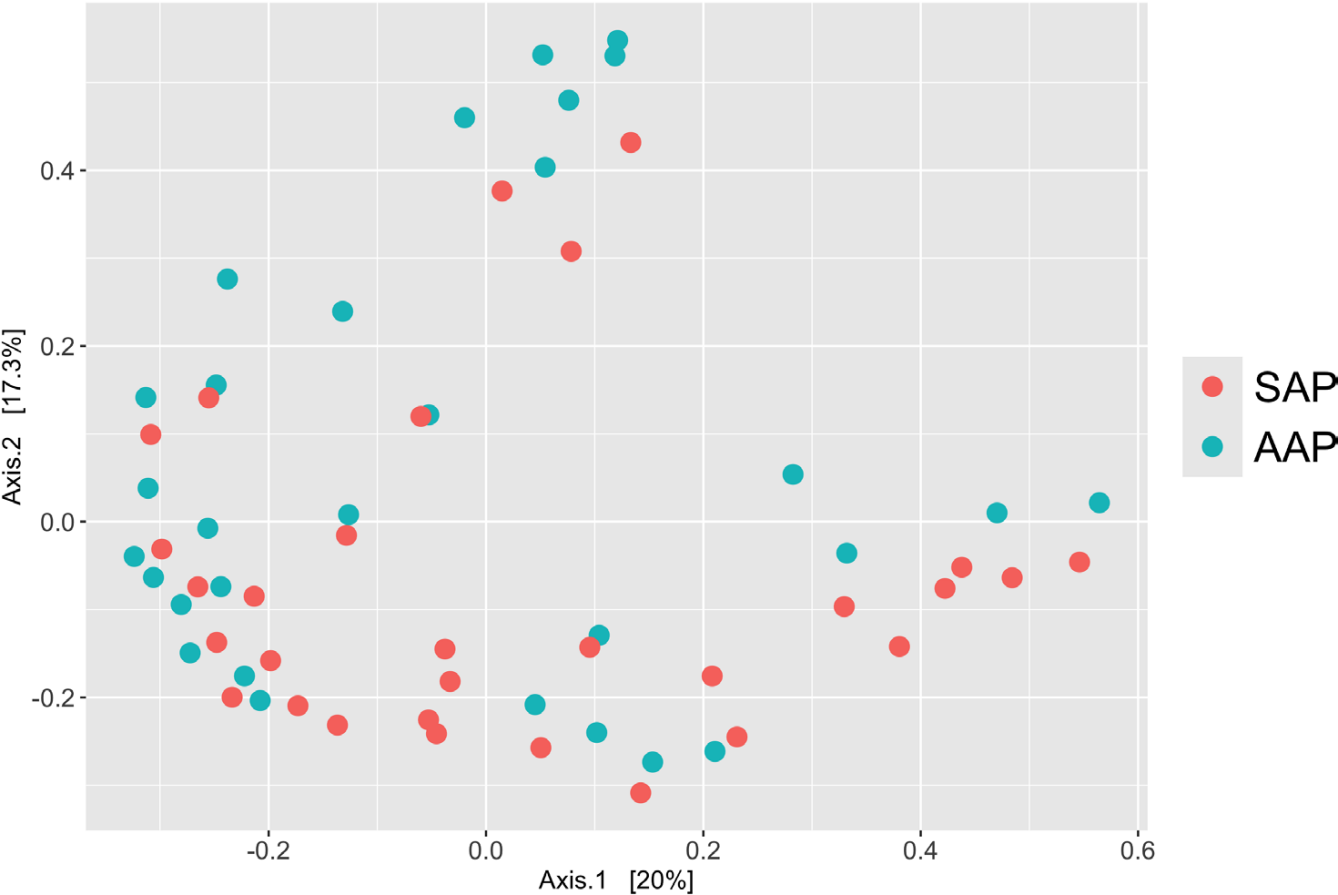
Genus level: Principal coordinate analysis (PCoA) based on Bray–Curtis dissimilarity. Each dot represents a microbial sample taken from teeth diagnosed with either SAP (red, *n* = 30) or AAP (blue, *n* = 30).

In Figure 2, a principal coordinate analysis based on Bray‐Curtis‐Dissimilarity is presented. No statistically significant differences in community composition between groups on genus level were observed (*p* = 0.085; *R* ^2^ = 0.02695).

**FIGURE 3:**
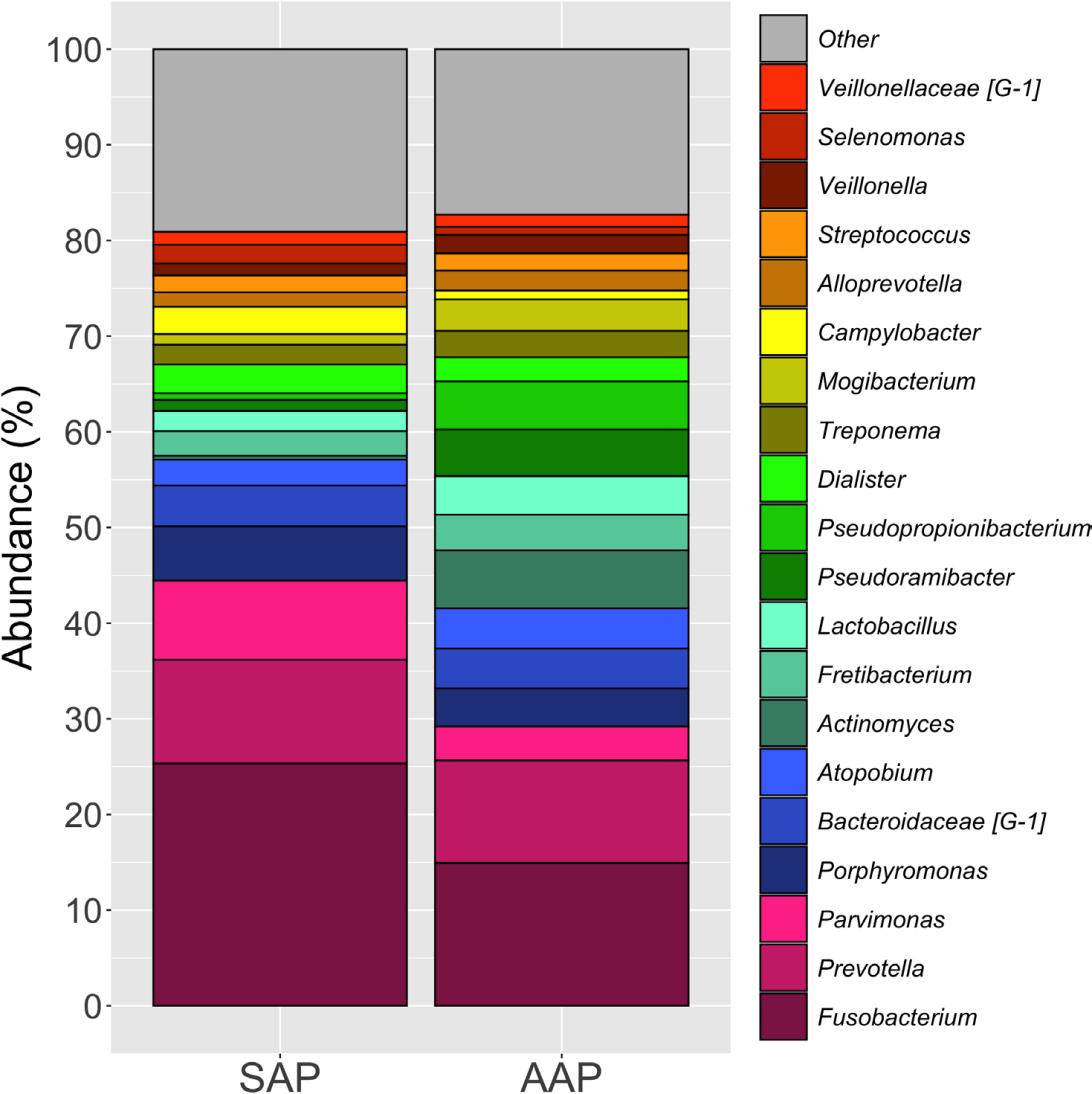
Genus composition of microbial root canal samples. The graphs show the mean relative abundance of genera according to group SAP (*n* = 30) or AAP (*n* = 30). The top 20 most abundant genera are displayed, with all others grouped together.

**FIGURE 4:**
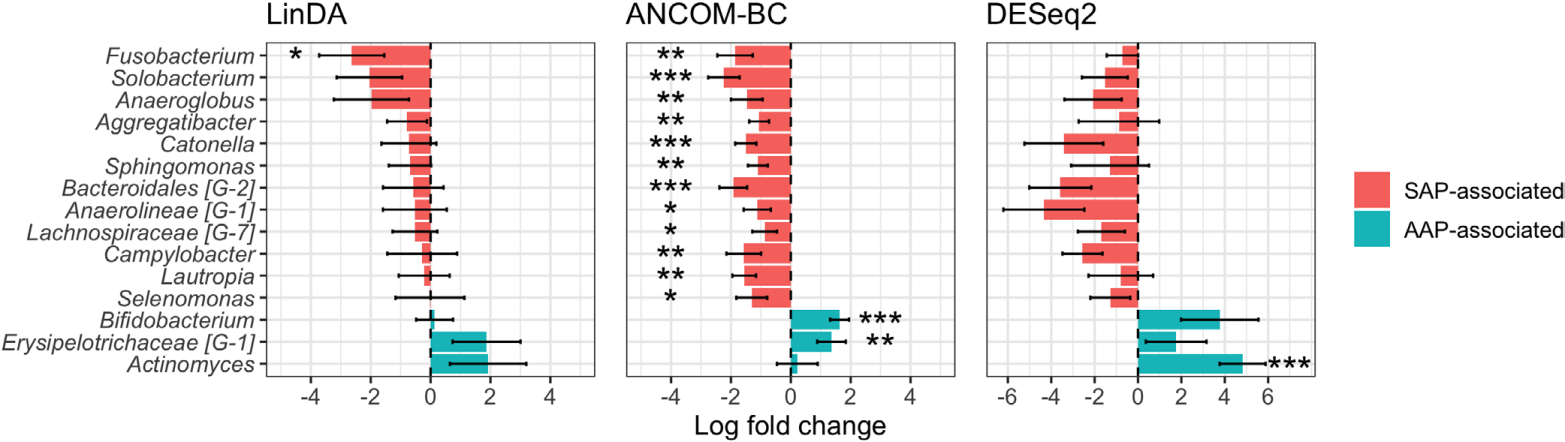
Differential abundance analysis of endodontic microbiome on genus level. Two‐group comparisons of symptomatic apical periodontitis (SAP, *n* = 30) and asymptomatic apical periodontitis (AAP, *n* = 30). Data are represented by effect size (log fold change) and standard error of log fold change bars (two‐sided) derived from the LinDA, ANCOM‐BC2, and DESeq2 models, respectively. SAP‐associated genera are coloured in red and AAP‐associated genera are coloured in green. All effect sizes with adjusted *p* < 0.05 are indicated, *significant at 5% level of significance; **significant at 1% level of significance; ***significant at 0.1% level of significance. Only genera with adjusted *p* < 0.05 are displayed.

There were in total 121 genera in the sample data set. SAP and AAP groups shared 106 of them. The top 20 most abundant genera with all others grouped together are displayed in Figure 3. The prevalence and mean relative abundance of each genus were calculated according to the groups (Table S4). The differential abundance analysis was performed on a dataset comprising 66 genera after prevalence filtering. The differential abundance analyses indicated a total of 15 genera to be differently abundant. The LinDA method indicated *Fusobacterium*, the ANCOM‐BC method indicated *Fusobacterium*, *Solobacterium*, *Anaeroglobus*, *Aggregatibacter*, *Catonella*, *Sphingomonas*, *Bacteroidales [G‐2]*, *Anaerolineae [G‐1]*, *Lachnospiraceae [G‐7]*, *Campylobacter*, *Lautropia*, *Selenomonas, Bifidobacterium, Erysipelotrichaceae*, and the DESeq2 method indicated *Actinomyces* to be differently abundant. The directions of the log fold changes were consistent over all differential abundance methods. Among the highly abundant genera, *Fusobacterium* was indicated to be more abundant in SAP samples whereas *Actinomyces* were more abundant in AAP samples (Figure 4).

### 3.2 ASV Level

A principal coordinate analysis based on Bray‐Curtis‐Dissimilarity is shown in Figure S2 without statistically significant difference in community composition between groups on ASV‐level (*p* = 0.15; *R* ^2^ = 0.02059). The top 20 most abundant ASVs are displayed in Figure S3 with all others grouped together. ASVs associated with *Fusobacterium* species are proportionally more abundant in SAP.

### 3.3 Controls

As reported in a previous study, irrelevant contamination was observed. DNA extraction controls contained 23.2 ± 11.8 ASVs (1830 ± 2410 reads), library preparation controls 10.2 ± 6.94 ASVs (177 ± 139 reads), and tris buffer controls containing new sterile NiTi instruments 29.3 ± 5.69 ASVs (1540 ± 1620 reads), respectively (Donnermeyer et al. 2025).

## 4 Discussion

This cross‐sectional clinical study compared the endodontic microbiome of symptomatic and asymptomatic cases of apical periodontitis. Concerning alpha diversity, no differences in richness and diversity (Shannon diversity index) were observed either on genus or ASV level. No significant differences were observed on either genus or ASV level for beta diversity comparing SAP and AAP samples. Hence, the null hypothesis was accepted. However, the differential abundance analyses revealed certain genera that significantly differed in their relative abundance between AAP and SAP.

Although overall microbial composition was largely similar between symptomatic and asymptomatic cases, the genus *Fusobacterium* emerged as more abundant in symptomatic infections among the highly prevalent genera. This finding corroborates the hypothesis that the expression of symptoms deriving from apical inflammation after root canal infection depends on the constitution of the endodontic microbiome (Sakamoto et al. 2006; Santos et al. 2011; Georgiou et al. 2023). Though such a difference was observed afore, the heterogeneity of study designs, detection techniques and sequencing depths did not allow to observe specific patterns. In contrast, newer studies could not relate the composition of the endodontic microbiome to clinical symptoms (Ordinola‐Zapata et al. 2023; Schuweiler et al. 2024). Likewise, the genera present in the microbiomes were similar in the groups of the present study, but differential abundance analysis was able to identify differences in the percentual composition.

In the past, one approach to investigate differences between microbiomes associated with different manifestations of apical inflammation was to identify more and more species present in root canal infections. Though more than 500 bacterial species were identified, most of these are low‐abundant and the endodontic microbiome is dominated by the same genera throughout different studies (Mominkhan et al. 2025). Accordingly, in the present study, nine genera were identified associated solely with SAP and six genera were solely associated with AAP, respectively. While such differences could be observed, these genera accounted only for a small proportion and relative abundance of bacteria observed in the specimens. Overall, these 15 unshared genera did neither represent a substantial number of samples (low prevalence) within their group nor did they represent a substantial proportion of reads (low relative abundance) within the positive samples (Table S4). For example, a presumed contaminant genus such as *Acinetobacter* (Donnermeyer et al. 2025) was found even similarly or more prevalent and abundant. Consequently, symptom expression is unlikely to be driven by a specific infection of the root canal system and the significance of these unshared genera should be interpreted with caution.

A more promising approach is to investigate the most common and dominating bacteria in the composition of the endodontic microbiome. Shifts and altered interplay in the bacterial communities could lead to changes in the expression of symptoms (Siqueira and Rocas 2009). Regarding the results of the present study, the predominance of *Fusobacterium* on genus level (Figure 3) and *Fusobacterium* species on ASV level (Figure S3) associated with SAP becomes evident. Such an interconnection between *Fusobacterium* species and acute clinical symptoms based on culture techniques was already reported (Gomes et al. 2021; Chavez de Paz Villanueva 2002). *Fusobacterium* species are generally regarded as virulent anaerobic gram‐negative species associated with endodontic or periodontal disease. In periodontal disease, *Fusobacterium nucleatum* is associated with progression of attachment loss (Signat et al. 2011; Chen et al. 2022). According to a recent meta‐analysis, *Fusobacterium* genus was widely detected in symptomatic as well as in asymptomatic root canal infections but not associated with a specific expression of symptoms (Alhadainy et al. 2023). However, the result of that meta‐analysis reflects the heterogeneity of the studies and is according to the authors associated with high risk of bias and a very low level of certainty (Alhadainy et al. 2023). Another meta‐analysis concluded that the role of the order *Fusobacteriales* on symptoms remains uncertain, but identified *Spirochetes* as associated with SAP (Aboushadi et al. 2024). Therefore, their results could also be interpreted as a demand for more highly qualitative studies with deeper differentiation of genus or species level rather than a conclusive statement on the role of *Fusobacterium* species in endodontic infections. In the differential abundance analysis, nine genera presented differently abundant between SAP and AAP. Applying different methods on the 66 most highly prevalent genera, *Fusobacterium* was indicated to be more abundant in SAP samples whereas among other *Actinomyces* was more abundant in AAP samples. No comparable studies or analyses are available at this point, again reflecting the need for more depth and quality in the analysis of endodontic microbiomes. At least, a recent study reported *Actinobacteria* as one of the phyla dominating the microbiome of AAP (Amaral et al. 2022). The present results focus on primary root canal infection, but could also apply to secondary infections of the root canal system, since recent studies indicated similar composition of primary and secondary infections, though primary infections presented more diverse (Ordinola‐Zapata et al. 2023). Accordingly, no differences of the microbiome related to the clinical symptoms of primary or secondary infections were observed (Ordinola‐Zapata et al. 2023). In both primary and secondary root canal infections, a high prevalence of *F. nucleatum* was reported, with a noted association with AAP symptoms (Gomes et al. 2021; Perez‐Carrasco et al. 2023). However, the results regarding the similarity in primary and secondary infections are inconsistent and therefore should be interpreted cautiously, as a recent review concluded that differences exist between primary and secondary infections (Siqueira et al. 2024).

The present results clearly emphasise the role of *Fusobacterium* in symptomatic endodontic infections. Microorganisms possessing virulence factors such as *Fusobacterium* (Arias‐Moliz et al. 2024) can shape the composition of microbial communities, triggering inflammation and ultimately contributing to pain and tenderness to percussion (Alhadainy et al. 2023). Although *Fusobacterium* may be present in relatively small amounts, it can enhance the pathogenicity of many other bacteria (George et al. 2016). *F. nucleatum* enhanced the adhesion and invasion of other oral bacteria in an in vitro study and, when coinfecting with them, can suppress host immune responses to promote their persistent growth (Li et al. 2015). However, it remains unclear whether these harmful microbial partnerships form early in infection or emerge later as environmental conditions change (Rocas et al. 2011). The endodontic microbiome interacts with the host immune defence. The expression of host immune response related proteins could be linked to the clinical state of the apical periodontitis (Loureiro et al. 2021). A bidirectional interaction between the root canal microbiome and the host immune defence was postulated as different systemic inflammatory profiles were observed depending on the clinical expression of apical periodontitis (Georgiou et al. 2023).

Gene sequencing analysis has become a widespread standard approach for analyses of microbiomes. However, gene sequencing has evolved over the recent years allowing deeper understanding of the microbiome. A drawback of these developments and the evolving statistical analyses is that comparison between studies has limitations. Different sequencing techniques being applied lead to a heterogeneity of results presented in the literature (Siqueira and Rocas 2022b, 2022a). Besides, the effect of geographical disparities is difficult to estimate given the limited number of available studies. Still, significant differences in the prevalence were reported between different countries (Arias‐Moliz, Ordinola‐Zapata, et al. 2024). Moreover, interindividual variability hampers to identify specific bacterial species or patterns in studies with too few samples (Siqueira and Rocas 2022b). In comparison, the sample size is a major strength of the present study that could overcome limitations of studies with smaller sample sizes. Moreover, the use of a contemporary sequencing technique allows for reliable analysis on the genus level. Small sample sizes are even more critical in cross‐sectional studies, as this study model is prone to lack representativeness for a larger population. In addition to the limited evidence about disease specific bacterial communities, interindividual variations due to tooth type, periodontal and restorative status and patient‐related variables such as age and systemic conditions could affect the composition of the endodontic microbiome and therefore should be matter of future research. Besides individual factors, systemic medications, especially antibiotics could interfere with the endodontic microbiome. No studies have yet addressed this topic and hence a clear suggestion for a minimum duration between intake and sampling is missing and the decision becomes a weighing between preferably long periods and reliability of patients' testimonies. A study of the salivary oral microbiome reported greater resistance to antibiotic perturbation than that observed in the gut microbiome (Zaura et al. 2015). Given the likely little effect of systemic antibiotics on biofilms in a space separated from saliva and blood flow, the decision was made for a short, but reliable period in the present study. However, future research is needed to address this gap in knowledge. Furthermore, the number of studies addressing and comparing microbiomes in primary acute or asymptomatic infections, especially with sufficient sample sizes is scarce. This highlights the need for more highly qualitative studies to gain insight into the endodontic microbiome considering the clinical presentation of the disease. Illumina‐based amplicon sequencing has evolved to a standard technique over the recent years and allows for a safe interpretation of genus level. Denoising tools enabled the inference of exact ASVs which provided better taxonomic resolution and better comparability between experiments than, for example, OTUs (Callahan, Mcmurdie, et al. 2016). ASV level analysis is also highly sensitive to technical aspects including sequencing errors, batch effects, and other method inherent characteristics. Therefore, the biological meaning of ASV level results should be interpreted with proper caution. However, more advanced techniques full‐length 16S rRNA sequencing techniques have become available recently and show potential for a more comprehensive analysis on species level (Agustinho et al. 2024). This could overcome inherent limitations of the Illumina MiSeq method. While Illumina MiSeq provides highly accurate short reads, its limited read length restricts taxonomic resolution, making it difficult to reliably distinguish closely related species using short ASVs alone. Hence, differential abundance analysis was conducted on genus level in the present study to ensure greater validity of the results.

In a wider context, the results of the present study indicate that no specific microorganisms are responsible for the expression of symptoms of endodontic origin but that the microbiome composition with its immunological characteristics likely induce a corresponding immune response of the host. Certain micro‐environmental factors may promote a more immunologically aggressive multispecies community composition, which can lead to acute inflammation in the periradicular tissues (Siqueira and Rocas 2009). However, it is still uncertain whether these highly virulent communities emerge at the onset of infection or develop later due to environmental changes that alter the bacterial composition (Rocas et al. 2011) and no evidence exists how and why such alterations develop and what could initiate them, respectively, guiding to future research fields.

## 5 Conclusion

SAP and AAP were associated with diverse microbial communities, and no specific infection could be linked to a distinct clinical presentation of apical periodontitis. Nevertheless, compositional differences were observed between symptomatic and asymptomatic cases. These findings suggest that clinical symptoms are not attributable to individual microorganisms but could be influenced by shifts in the overall community structure and relative microbial composition.

## Author Contributions

David Donnermeyer: Conceptualization; Investigation; Methodology; Project administration; Validation; Visualisation; Roles/Writing – original draft; Edgar Schäfer: Supervision, Writing – review and editing. Daniel Hagenfeld: Formal analysis; Validation; Writing – review and editing. Benjamin Ehmke: Resources; Supervision, Writing – review and editing. Karola Prior: Methodology; Investigation. Dag Harmsen: Methodology. Madgalena Ibing: Investigation. Sebastian Bürklein: Investigation. Thomas Gerhard Wolf: Writing – review and editing. Johannes Matern: Data curation; Formal analysis; Software; Visualisation; Writing – review and editing.

## Funding

The authors have nothing to report.

## Conflicts of Interest

The authors declare no conflicts of interest.

## Supporting information


**Figure S1:** Rarefaction curves of (A) SAP samples (*n* = 62) and (B) AAP samples (*n* = 58).

**Figure S2:** ASV Level: Principal coordinate analysis (PCoA) based on Bray–Curtis dissimilarity. Each dot represents a microbial sample taken from teeth diagnosed with either SAP (red, *n* = 30) or AAP (blue, *n* = 30).

**Figure S3:** ASV composition of microbial root canal samples. The graphs show the mean relative abundance of ASVs according to group SAP (*n* = 30) or AAP (*n* = 30). The top 20 most abundant ASVs are displayed, with all others grouped together.

**Table S1:** Demographic values and patients' characteristics.

**Table S2:** Percentage of classified ASVs according to taxonomic rank.

**Table S3:** Alpha diversity parameters on ASV level.

**Table S4:** Prevalence and mean relative abundance on genus level in SAP and AAP specimens.

## Data Availability

The data that support the findings of this study are available from the corresponding author upon reasonable request.
