# Supplementary figures and images for: A Comparison of Endodontic Microbiomes Associated With Symptomatic and Asymptomatic Apical Periodontitis by Next‐Generation Sequencing
Source: Int Endod J. 2026 Mar 13;59(8):1608–18. doi: 10.1111/iej.70140 (PMC13373031; doi:10.1111/iej.70140)

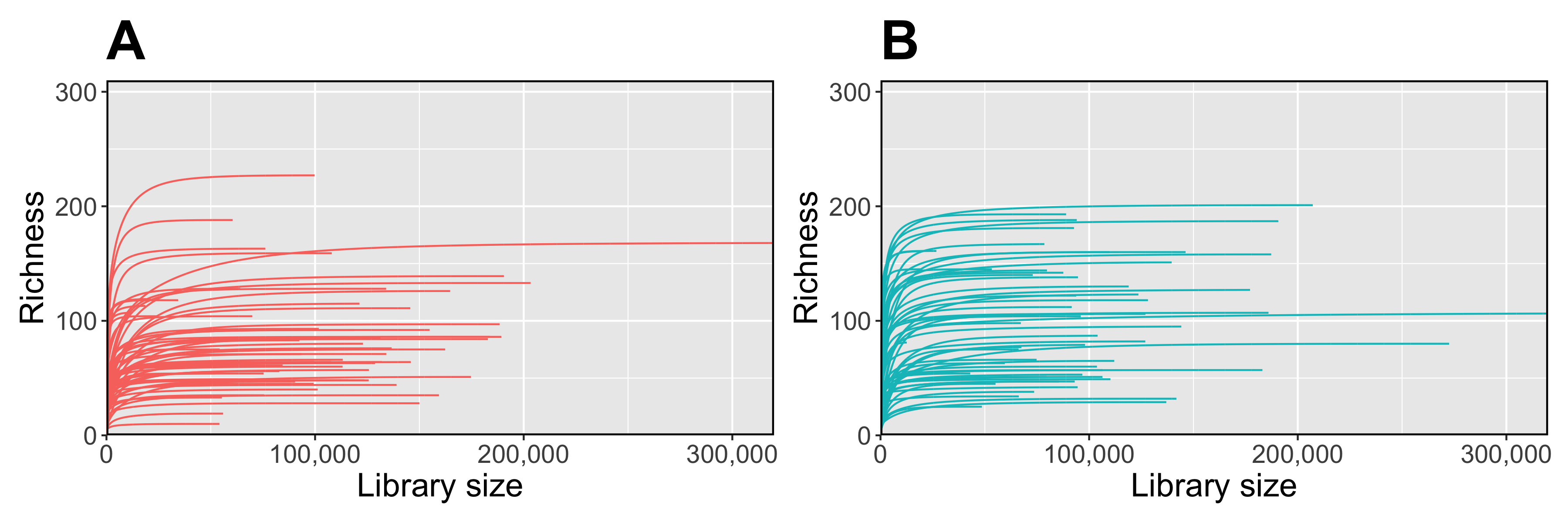

Supplement: Supplementary file 1 — Figure S1: Rarefaction curves of (A) SAP samples (n = 62) and (B) AAP samples (n = 58). [file IEJ-59-1608-s006.png]

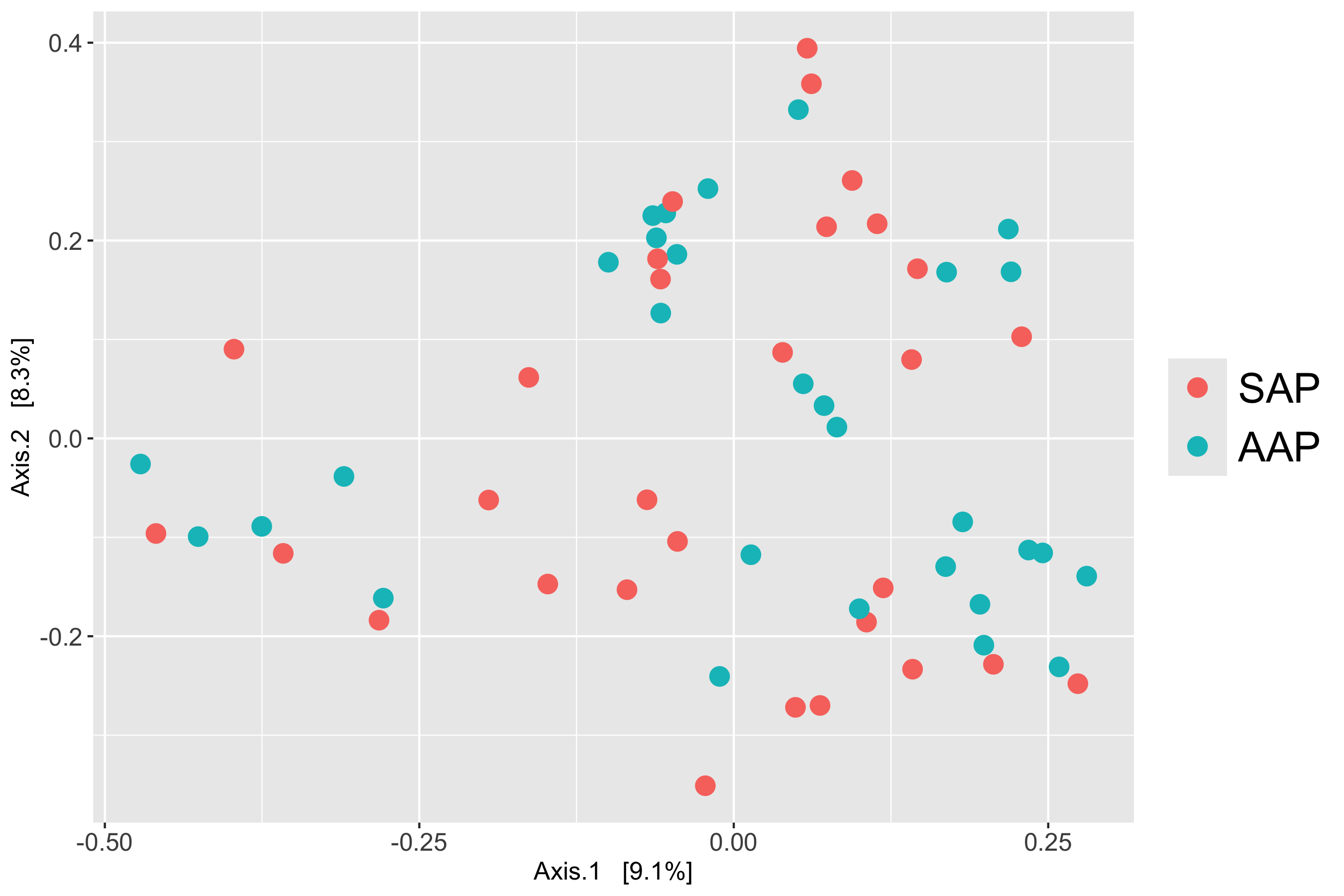

Supplement: Supplementary file 2 — Figure S2: ASV Level: Principal coordinate analysis (PCoA) based on Bray–Curtis dissimilarity. Each dot represents a microbial sample taken from teeth diagnosed with either SAP (red, n = 30) or AAP (blue, n = 30). [file IEJ-59-1608-s005.png]

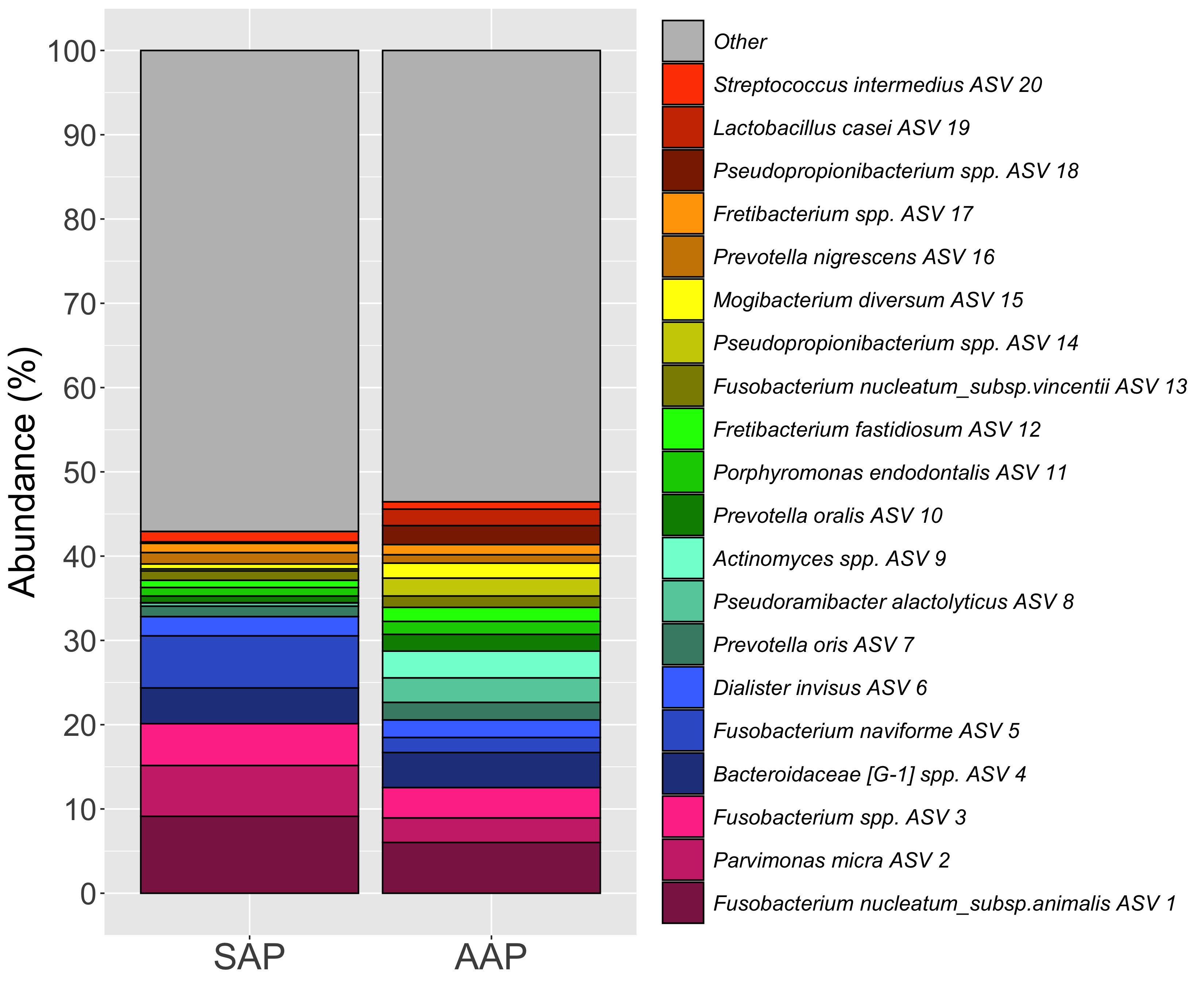

Supplement: Supplementary file 3 — Figure S3: ASV composition of microbial root canal samples. The graphs show the mean relative abundance of ASVs according to group SAP (n = 30) or AAP (n = 30). The top 20 most abundant ASVs are displayed, with all others grouped together. [file IEJ-59-1608-s002.png]
