# Supplementary material for: A Comparison of Endodontic Microbiomes Associated With Symptomatic and Asymptomatic Apical Periodontitis by Next‐Generation Sequencing
Source: Int Endod J. 2026 Mar 13;59(8):1608–18. doi: 10.1111/iej.70140 (PMC13373031; doi:10.1111/iej.70140)
Supplement: Supplementary file 4 — Table S1: Demographic values and patients' characteristics. [file IEJ-59-1608-s003.docx]

**Suppl. Table S1** Demographic values and patients’ characteristics

| **specimen no.** | **patient no.** | **group** | **gender** | **age** | **tooth** | **sensitivity to cold** | **tenderness to percussion** | **presence of sinus tract** |
| --- | --- | --- | --- | --- | --- | --- | --- | --- |
| 1 | 1 | SAP | female | 45 | 45 | negative | positive | negative |
| 2 | 2 | SAP | male | 67 | 35 | negative | positive | negative |
| 3 | 3 | SAP | female | 53 | 17 | negative | positive | negative |
| 4 | 4 | SAP | male | 27 | 46 | negative | positive | negative |
| 5 | 5 | SAP | female | 61 | 24 | negative | positive | negative |
| 6 | 6 | SAP | female | 22 | 25 | negative | positive | negative |
| 7 | 7 | SAP | female | 58 | 14 | negative | positive | negative |
| 8 | 8 | SAP | male | 42 | 15 | negative | positive | negative |
| 9 | 9 | SAP | male | 36 | 17 | negative | positive | negative |
| 10 | 10 | SAP | female | 58 | 33 | negative | positive | negative |
| 11 | 11 | SAP | male | 69 | 26 | negative | positive | negative |
| 12 | 12 | SAP | female | 59 | 33 | negative | positive | negative |
| 13 | 13 | SAP | female | 69 | 35 | negative | positive | negative |
| 14 | 14 | SAP | male | 56 | 21 | negative | positive | negative |
| 15 | 15 | SAP | female | 44 | 44 | negative | positive | negative |
| 16 | 16 | SAP | female | 55 | 14 | negative | positive | negative |
| 17 | 17 | SAP | male | 19 | 11 | negative | positive | negative |
| 18 | 18 | SAP | female | 33 | 16 | negative | positive | negative |
| 19 | 19 | SAP | female | 52 | 26 | negative | positive | negative |
| 20 | 20 | SAP | female | 34 | 22 | negative | positive | negative |
| 21 | 21 | SAP | male | 38 | 47 | negative | positive | negative |
| 22 | 22 | SAP | female | 61 | 24 | negative | positive | negative |
| 23 | 23 | SAP | female | 46 | 27 | negative | positive | negative |
| 24 | 24 | SAP | male | 71 | 46 | negative | positive | negative |
| 25 | 25 | SAP | male | 51 | 36 | negative | positive | negative |
| 26 | 26 | SAP | male | 65 | 46 | negative | positive | negative |
| 27 | 27 | SAP | female | 40 | 28 | negative | positive | negative |
| 28 | 28 | SAP | male | 63 | 36 | negative | positive | negative |
| 29 | 29 | SAP | female | 72 | 11 | negative | positive | negative |
| 30 | 30 | SAP | male | 72 | 27 | negative | positive | negative |
| 31 | 31 | AAP | female | 56 | 24 | negative | negative | negative |
| 32 | 32 | AAP | male | 60 | 27 | negative | negative | negative |
| 33 | 33 | AAP | male | 49 | 25 | negative | negative | negative |
| 34 | 34 | AAP | female | 63 | 16 | negative | negative | negative |
| 35 | 35 | AAP | female | 39 | 36 | negative | negative | negative |
| 36 | 36 | AAP | female | 34 | 26 | negative | negative | negative |
| 37 | 37 | AAP | male | 51 | 37 | negative | negative | negative |
| 38 | 38 | AAP | male | 45 | 11 | negative | negative | negative |
| 39 | 39 | AAP | male | 49 | 17 | negative | negative | negative |
| 40 | 40 | AAP | female | 40 | 22 | negative | negative | negative |
| 41 | 41 | AAP | female | 58 | 11 | negative | negative | negative |
| 42 | 42 | AAP | female | 63 | 44 | negative | negative | negative |
| 43 | 43 | AAP | male | 55 | 36 | negative | negative | negative |
| 44 | 44 | AAP | female | 59 | 25 | negative | negative | negative |
| 45 | 45 | AAP | female | 32 | 12 | negative | negative | negative |
| 46 | 46 | AAP | male | 56 | 13 | negative | negative | negative |
| 47 | 46 | AAP | male | 56 | 14 | negative | negative | negative |
| 48 | 47 | AAP | male | 45 | 16 | negative | negative | negative |
| 49 | 48 | AAP | male | 54 | 11 | negative | negative | negative |
| 50 | 49 | AAP | female | 62 | 24 | negative | negative | negative |
| 51 | 50 | AAP | female | 69 | 16 | negative | negative | negative |
| 52 | 51 | AAP | male | 66 | 44 | negative | negative | negative |
| 53 | 52 | AAP | male | 43 | 33 | negative | negative | negative |
| 54 | 53 | AAP | male | 68 | 27 | negative | negative | negative |
| 55 | 54 | AAP | male | 57 | 25 | negative | negative | negative |
| 56 | 55 | AAP | male | 25 | 46 | negative | negative | negative |
| 57 | 56 | AAP | female | 34 | 14 | negative | negative | negative |
| 58 | 57 | AAP | male | 71 | 41 | negative | negative | negative |
| 59 | 58 | AAP | female | 62 | 27 | negative | negative | negative |
| 60 | 59 | AAP | male | 73 | 47 | negative | negative | negative |
