# Supplementary material for: A Comparison of Endodontic Microbiomes Associated With Symptomatic and Asymptomatic Apical Periodontitis by Next‐Generation Sequencing
Source: Int Endod J. 2026 Mar 13;59(8):1608–18. doi: 10.1111/iej.70140 (PMC13373031; doi:10.1111/iej.70140)
Supplement: Supplementary file 5 — Table S2: Percentage of classified ASVs according to taxonomic rank. [file IEJ-59-1608-s004.docx]

**Suppl. Table S2** Percentage of classified ASVs according to taxonomic rank

| **Taxonomic rank** | **Classified ASVs (%)** |
| --- | --- |
| Kingdom | 100.00 |
| Phylum | 91.86 |
| Class | 90.62 |
| Order | 90.30 |
| Family | 89.88 |
| Genus | 89.39 |
| Species | 42.60 |
