# Supplementary material for: A Comparison of Endodontic Microbiomes Associated With Symptomatic and Asymptomatic Apical Periodontitis by Next‐Generation Sequencing
Source: Int Endod J. 2026 Mar 13;59(8):1608–18. doi: 10.1111/iej.70140 (PMC13373031; doi:10.1111/iej.70140)
Supplement: Supplementary file 6 — Table S3: Alpha diversity parameters on ASV level. [file IEJ-59-1608-s001.docx]

**Suppl. Table S3** Alpha diversity parameters on ASV level

|  | **SAP (N=30)** | **AAP (N=30)** | ***p*-value** |
| --- | --- | --- | --- |
| **Richness** |  |  |  |
| Mean (SD) | 83.5 (42.9) | 94.1 (44.1) | 0.359 |
| Median [Q1, Q3] | 81.5 [54.8, 113] | 95.0 [60.0, 114] |  |
| **Diversity** |  |  |  |
| Mean (SD) | 2.46 (0.773) | 2.43 (1.01) | 0.901 |
| Median [Q1, Q3] | 2.48 [2.11, 2.97] | 2.67 [1.68, 3.24] |  |

Richness, Number of observed genera; Diversity, Shannon diversity index. Unpaired Mann-Whitney U tests.
