# Supplementary material for: A Comparison of Endodontic Microbiomes Associated With Symptomatic and Asymptomatic Apical Periodontitis by Next‐Generation Sequencing
Source: Int Endod J. 2026 Mar 13;59(8):1608–18. doi: 10.1111/iej.70140 (PMC13373031; doi:10.1111/iej.70140)
Supplement: Supplementary file 7 — Table S4: Prevalence and mean relative abundance on genus level in SAP and AAP specimens. [file IEJ-59-1608-s007.docx]

**Suppl. Table S4** Prevalence and mean relative abundance on genus level in SAP and AAP specimens

|  | **SAP** | | **AAP** | |
| --- | --- | --- | --- | --- |
| **Genus** | **Prevalence**  **(%)** | **Abundance (mean, %)** | **Prevalence**  **(%)** | **Abundance (mean, %)** |
| Fusobacterium | 97 | 25.34 | 93 | 14.94 |
| Prevotella | 97 | 10.85 | 97 | 10.71 |
| Parvimonas | 90 | 8.26 | 80 | 3.56 |
| Porphyromonas | 73 | 5.68 | 80 | 3.98 |
| Bacteroidaceae [G-1] | 63 | 4.26 | 57 | 4.17 |
| Atopobium | 73 | 2.7 | 87 | 4.2 |
| Actinomyces | 63 | 0.42 | 83 | 6.06 |
| Fretibacterium | 87 | 2.57 | 83 | 3.71 |
| Lactobacillus | 20 | 2.1 | 30 | 4.04 |
| Pseudoramibacter | 50 | 1.15 | 57 | 4.88 |
| Pseudopropionibacterium | 30 | 0.71 | 33 | 5.02 |
| Dialister | 80 | 3 | 90 | 2.54 |
| Treponema | 80 | 2.06 | 80 | 2.74 |
| Mogibacterium | 63 | 1.11 | 73 | 3.3 |
| Campylobacter | 70 | 2.86 | 80 | 0.92 |
| Alloprevotella | 53 | 1.49 | 70 | 2.1 |
| Streptococcus | 70 | 1.77 | 83 | 1.79 |
| Veillonella | 80 | 1.24 | 83 | 1.94 |
| Selenomonas | 67 | 1.96 | 77 | 0.84 |
| Veillonellaceae [G-1] | 53 | 1.35 | 70 | 1.28 |
| Anaeroglobus | 53 | 1.69 | 43 | 0.63 |
| Peptostreptococcaceae [XI][G-1] | 73 | 1.58 | 60 | 0.68 |
| Tannerella | 73 | 0.68 | 73 | 1.54 |
| Peptostreptococcus | 40 | 1.33 | 43 | 0.65 |
| Filifactor | 27 | 1.3 | 33 | 0.62 |
| Peptostreptococcaceae [XI][G-6] | 43 | 0.58 | 60 | 1.3 |
| Peptostreptococcaceae [XI][G-9] | 33 | 1.36 | 40 | 0.47 |
| Acidipropionibacterium | 30 | 0.45 | 33 | 1.17 |
| Oribacterium | 60 | 0.82 | 70 | 0.78 |
| Bacteroidetes [G-3] | 23 | 0.39 | 50 | 1.05 |
| Bacteroides | 17 | 1.41 | 3 | 0.01 |
| Erysipelotrichaceae [G-1] | 37 | 0.14 | 47 | 0.96 |
| Solobacterium | 63 | 0.82 | 57 | 0.24 |
| Megasphaera | 33 | 0.57 | 20 | 0.34 |
| Stomatobaculum | 27 | 0.32 | 30 | 0.56 |
| Gemella | 37 | 0.13 | 40 | 0.67 |
| Bacteroidetes [G-5] | 7 | 0.15 | 30 | 0.64 |
| Anaerolineae [G-1] | 23 | 0.34 | 23 | 0.37 |
| Pyramidobacter | 7 | 0.68 | 3 | 0 |
| Capnocytophaga | 47 | 0.46 | 53 | 0.14 |
| Olsenella | 47 | 0.18 | 50 | 0.38 |
| Cryptobacterium | 7 | 0.3 | 17 | 0.26 |
| Bacteroidales [G-2] | 33 | 0.33 | 40 | 0.17 |
| Slackia | 50 | 0.18 | 60 | 0.3 |
| Peptostreptococcaceae [XI][G-4] | 40 | 0.18 | 50 | 0.22 |
| Mycoplasma | 37 | 0.15 | 40 | 0.23 |
| Shuttleworthia | 40 | 0.18 | 33 | 0.18 |
| Neisseria | 37 | 0.25 | 50 | 0.07 |
| Cutibacterium | 47 | 0.07 | 50 | 0.24 |
| Leptotrichia | 37 | 0.19 | 47 | 0.12 |
| Corynebacterium | 30 | 0.2 | 23 | 0.02 |
| Lachnospiraceae [G-2] | 0 | 0 | 10 | 0.21 |
| Rothia | 37 | 0.1 | 50 | 0.11 |
| Lautropia | 27 | 0.1 | 37 | 0.07 |
| Lachnoanaerobaculum | 27 | 0.13 | 30 | 0.03 |
| Desulfobulbus | 23 | 0.05 | 23 | 0.12 |
| Eggerthia | 17 | 0.11 | 13 | 0.05 |
| Saccharibacteria (TM7) [G-1] | 23 | 0.05 | 43 | 0.11 |
| Peptostreptococcaceae [XI][G-5] | 0 | 0 | 20 | 0.15 |
| Lachnospiraceae [G-7] | 47 | 0.03 | 47 | 0.1 |
| Catonella | 27 | 0.11 | 23 | 0.02 |
| Granulicatella | 33 | 0.06 | 33 | 0.07 |
| Johnsonella | 3 | 0.11 | 3 | 0 |
| Haemophilus | 40 | 0.04 | 43 | 0.07 |
| Eikenella | 17 | 0.02 | 27 | 0.09 |
| Bulleidia | 3 | 0 | 23 | 0.11 |
| Sneathia | 0 | 0 | 7 | 0.1 |
| Mitsuokella | 33 | 0.05 | 20 | 0.05 |
| Peptostreptococcaceae [XI][G-7] | 13 | 0.01 | 17 | 0.09 |
| Peptostreptococcaceae [XI][G-2] | 17 | 0.03 | 47 | 0.07 |
| Bifidobacterium | 23 | 0.01 | 23 | 0.09 |
| Acinetobacter | 17 | 0.01 | 17 | 0.08 |
| Peptoniphilus | 10 | 0.03 | 17 | 0.05 |
| Parascardovia | 10 | 0.01 | 13 | 0.07 |
| Aggregatibacter | 23 | 0.07 | 17 | 0 |
| Lawsonella | 13 | 0.03 | 27 | 0.05 |
| Bacteroidetes [G-7] | 3 | 0.06 | 7 | 0.01 |
| Lachnospiraceae [G-8] | 13 | 0.02 | 20 | 0.04 |
| Cardiobacterium | 7 | 0.06 | 3 | 0 |
| Sphingomonas | 27 | 0.05 | 23 | 0.01 |
| Enterococcus | 3 | 0.03 | 7 | 0.02 |
| Kingella | 7 | 0.04 | 30 | 0.02 |
| Peptostreptococcaceae [XI][G-3] | 10 | 0.01 | 23 | 0.04 |
| Clostridiales [F-1][G-1] | 10 | 0.01 | 20 | 0.04 |
| Peptidiphaga | 10 | 0.04 | 23 | 0 |
| Desulfovibrio | 3 | 0 | 7 | 0.03 |
| Lachnospiraceae [G-3] | 13 | 0.02 | 7 | 0.01 |
| Staphylococcus | 30 | 0.01 | 30 | 0.01 |
| Saccharibacteria (TM7) [G-3] | 10 | 0.02 | 10 | 0.01 |
| Rhodocyclus | 17 | 0.02 | 3 | 0 |
| Scardovia | 17 | 0 | 17 | 0.02 |
| Peptoniphilaceae [G-1] | 3 | 0.02 | 0 | 0 |
| Achromobacter | 7 | 0.01 | 3 | 0.01 |
| Lactococcus | 3 | 0 | 3 | 0.02 |
| Abiotrophia | 13 | 0.01 | 30 | 0.01 |
| Bergeyella | 20 | 0.01 | 17 | 0.01 |
| Flavitalea | 13 | 0.01 | 0 | 0 |
| Desulfomicrobium | 3 | 0.01 | 0 | 0 |
| Saccharibacteria (TM7) [G-5] | 13 | 0 | 27 | 0.01 |
| Absconditabacteria (SR1) [G-1] | 7 | 0.01 | 3 | 0 |
| Acidovorax | 17 | 0.01 | 3 | 0 |
| Afipia | 27 | 0.01 | 7 | 0 |
| Bradyrhizobium | 3 | 0.01 | 0 | 0 |
| Porphyrobacter | 7 | 0.01 | 3 | 0 |
| Butyrivibrio | 3 | 0.01 | 3 | 0 |
| Bacteroidetes [G-6] | 3 | 0.01 | 0 | 0 |
| Bosea | 17 | 0.01 | 20 | 0 |
| Peptococcus | 7 | 0 | 17 | 0 |
| Mollicutes [G-2] | 0 | 0 | 7 | 0.01 |
| Saccharibacteria (TM7) [G-6] | 3 | 0 | 0 | 0 |
| Ruminococcaceae [G-1] | 13 | 0 | 3 | 0 |
| Novosphingobium | 7 | 0 | 3 | 0 |
| Moraxella | 20 | 0 | 3 | 0 |
| Gracilibacteria (GN02) [G-2] | 3 | 0 | 0 | 0 |
| Ruminococcaceae [G-2] | 7 | 0 | 3 | 0 |
| Gracilibacteria (GN02) [G-1] | 3 | 0 | 3 | 0 |
| Jonquetella | 0 | 0 | 3 | 0 |
| Enterobacter | 3 | 0 | 3 | 0 |
| Saccharibacteria (TM7) [G-4] | 3 | 0 | 0 | 0 |
| Saccharibacteria (TM7) [G-8] | 3 | 0 | 0 | 0 |
| Mollicutes [G-1] | 0 | 0 | 3 | 0 |
